# Quaternion-Based Unscented Kalman Filter for Accurate Indoor Heading Estimation Using Wearable Multi-Sensor System

**DOI:** 10.3390/s150510872

**Published:** 2015-05-07

**Authors:** Xuebing Yuan, Shuai Yu, Shengzhi Zhang, Guoping Wang, Sheng Liu

**Affiliations:** 1 School of Mechanical Science and Engineering, Huazhong University of Science and Technology, 1037 Luoyu Road, Wuhan 430074, China; E-Mails: xuebing_yuan@hust.edu.cn (X.Y.); yushuai91@hust.edu.cn (S.Y.); zhangshengzhi@hust.edu.cn (S.Z.); 2 School of Power and Mechanical Engineering, Wuhan University, 8 East Lake South Road, Wuhan 430072, China; E-Mail: guopingwang@whu.edu.cn

**Keywords:** quaternion, unscented Kalman filter, heading estimation, indoor navigation, wearable, multi-sensor

## Abstract

Inertial navigation based on micro-electromechanical system (MEMS) inertial measurement units (IMUs) has attracted numerous researchers due to its high reliability and independence. The heading estimation, as one of the most important parts of inertial navigation, has been a research focus in this field. Heading estimation using magnetometers is perturbed by magnetic disturbances, such as indoor concrete structures and electronic equipment. The MEMS gyroscope is also used for heading estimation. However, the accuracy of gyroscope is unreliable with time. In this paper, a wearable multi-sensor system has been designed to obtain the high-accuracy indoor heading estimation, according to a quaternion-based unscented Kalman filter (UKF) algorithm. The proposed multi-sensor system including one three-axis accelerometer, three single-axis gyroscopes, one three-axis magnetometer and one microprocessor minimizes the size and cost. The wearable multi-sensor system was fixed on waist of pedestrian and the quadrotor unmanned aerial vehicle (UAV) for heading estimation experiments in our college building. The results show that the mean heading estimation errors are less 10° and 5° to multi-sensor system fixed on waist of pedestrian and the quadrotor UAV, respectively, compared to the reference path.

## 1. Introduction

Autonomous indoor navigation is increasingly popular for pedestrians, mobile robots, and unmanned aerial vehicles (UAVs). The applications are not only for military purposes, but also for civil ones. The Global Position System (GPS) has a wide range of applications for navigation in land-, sea- and aviation-based fields, however, weak reception of GPS signals poses additional challenges to self-localization in indoor environments [1,2]. Therefore, it is necessary to explore the new positioning methods with better quality. For now, various techniques have been developed, such as Ultra-WideBand (UWB) communication [3], wireless local area network (WLAN) [4,5], WiFi-based [6,7,8], camera-based [9,10], ultrasonic sensors [11,12], laser-based [13], radio frequency identification (RFID) [14,15] and strapdown inertial navigation [16,17,18]. Among these techniques, inertial measurement unit (IMU)-based navigation is more competitive, owing to its independence of the existing infrastructures in buildings. This independence feature of IMU is particularly important for emergencies where there is no power supply in a building. Besides, it is convenient for strapdown inertial navigation to combine with GPS as an indoor-outdoor seamless navigation solution. The rapid development of micro-electro-mechanical system (MEMS) technology has accelerated the progress of IMUs, which have become low cost, small size, and low power consumption in recent years. However, the performance, e.g., long-term and short-term accuracy, of these low-cost MEMS IMU is limited by the complicated environment [19,20].

Accurate heading estimation plays a significant role in strapdown inertial navigation in indoor environments. The most common method is the magnetometer-based heading solution, which has been widely reported in [21,22,23,24], nevertheless, magnetometers are easily perturbed by magnetic disturbances from indoor iron structures and electronic equipment. Compensation of magnetic disturbances and magnetic perturbation detection methods for improving heading estimation are respectively demonstrated in [25,26]. With the current state of the art of MEMS technology, the accuracy of gyroscopes is not good enough for deriving heading estimation over longer terms because of their large biases and scale factors [27,28]. An effective method of minimizing drift in the heading estimation that relies solely on integration of rate signals from a gyro was examined to meet the challenges of making low-cost MEMS gyroscopes for the precise self-localization of indoor mobile robots [28,29]. Ali *et al.* proposed to use a Kalman filter algorithm of fusing low-cost MEMS gyroscope and magnetometer in a smartphone to obtain heading estimation with quaternion mechanization [30]. All the above works are inspirational, but regrettably, none of them has met the great challenge of heading estimation for complex indoor environments and moving carriers, even if the magnetometer calibration and restraining of gyroscope drift have been carried out.

A quaternion-based extended Kalman filter (EKF) algorithm has been proposed to improve heading determination with handheld IMUs in experiment and theory [31]. An integration algorithm using EKF was employed to reduce heading drift with a commercial IMU mounted on a shoe in indoor spaces [32]. Although these studies could offer effective heading estimations for indoor navigation, the linearization steps of EKF with the Jacobean matrix in attitude determination were not stable and accurate enough. In order to accommodate highly non-linear kinematics models related to the attitude estimation, the unscented Kalman filter (UKF) has been proved to be better solution than EKF, owing to the sigma-point approximation used in UKF [33,34]. The UKF in space applications had more robustness and accuracy than the EKF as shown in [35]. Although computational cost was a little higher than with EKF, there were new sigma-points algorithms aiming to reduce this cost in attitude determination [36]. However, those studies of UKF for attitude determination mainly focused on outdoor applications, and indoor context is equally important and more complicated. The quaternion-based Kalman filter was designed in [37] to for human body motion tracking, and the feasibility of real time human body motion tracking was validated. A novel quaternion Kalman filter was presented in [38], and the proposed filter succeeded in the particular case of high initial estimation errors whereas EKF fails to converge. A quaternion based EKF was developed for determining the orientation of a rigid body [39] and a quaternion-based indirect Kalman filter structure was used to obtain adaptive estimation of external acceleration [40]. However, the nonlinear estimations for attitude solution of the above works are weaker than UKF, which was demonstrated to be a powerful nonlinear estimation technique and had been shown to be a superior alternative to the EKF in a variety of applications [41].

To meet the requirements of accurate indoor heading estimation using a low-cost MEMS strapdown inertial navigation system, an effective method of quaternion-based UKF algorithm has been proposed. The quaternion-based UKF algorithm has the following advantages over the general Kalman filter and EKF [27,28,29,30,31,32,33,34,35,36,42,43,44,45,46]: (1) The quaternion-based orientation of an object has no singularities when the pitch angle passes through ±*π*/2 than Euler angles or Direction Cosine Matrix (DCM); (2) Quaternion-based matrix transformation has higher computational efficiency than Euler angles and DCM, and it is more suitable for our low cost MEMS multi-sensor system; (3) EKF models are linearized through a first order Taylor series expansion of the process/measurement models around the current state estimate, meanwhile, Jacobian matrix computation is quite complex; but UKF is second order approximation, which has capability of dealing with large and small attitude errors.

In this paper, a miniature wearable multi-sensor system is designed for accurate indoor heading estimation applied in mobile robots, indoor UAV and pedestrian navigation. The multi-sensor system is composed of low-cost MEMS devices, including one three-axis accelerometer, three single-axis gyroscopes, one three-axis magnetometer, and one microprocessor. The proposed quaternion-based UKF algorithm can make use of the complementary features of gyroscopes not affected by magnetic disturbance and magnetometers without long-term drift for heading estimation. The experiments are carried out with a multi-sensor system fixed on the waist of pedestrians and a quadrotor UAV.

This paper is organized as follows: in Section 2, the wearable multi-sensor system is described. The heading estimation multi-sensor system is discussed in Section 3. In Section 4 the proposed method comprising quaternion-based UKF algorithm is described in detail. Analysis of both experiments and results is given in Section 5. Finally, the conclusions and future work are summarized in Section 6.

## 2. Wearable Multi-Sensor System

**Figure 1:**
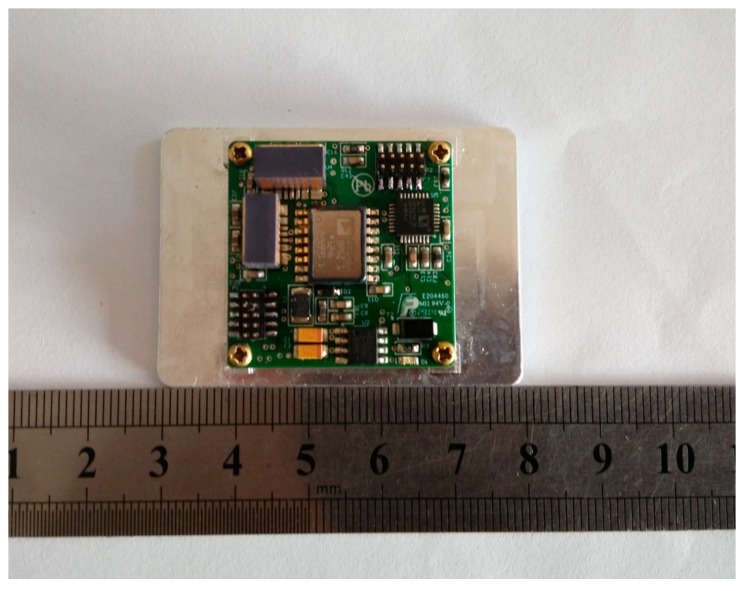
Wearable multi-sensor system designed by our lab.

The wearable multi-sensor system is a miniature strapdown inertial navigation system designed in our lab to satisfy the needs of indoor navigation for mobile robots, UAVs and pedestrians. The multi-sensor system is composed of one three-axis accelerometer, three single-axis gyroscopes and one magnetometer and one microprocessor, as shown in Figure 1, and its small size is 32 × 32 × 15 mm. In fact, our multi-sensor system can be regarded as a combination of an IMU and magnetometer. The accelerometer uses an ADXL312 made by Analog Devices Inc. (Norwood, MA, USA), and has a measurement range of ±12 g and high resolution of 2.9 mg/LSB with I^2^C/SPI bus for digital output. The ADXRS453 made by Analog Devices Inc. is selected for the three single-axis gyroscopes, and it is intended for industrial applications with its features of ±300°/s angular rate sensing and 16°/h bias instability. The three-axis magnetometer uses the HMC1053 from Honeywell Corp. (Morristown, NJ, USA), with a measurement range of ±6 gauss and resolution of 0.12 milligauss. We use the STM32F407 as our microprocessor, and it is based on the high-performance ARM Corte-M4 32-bit RISC core operating at a frequency of 168 MHz and 1 Mbyte of flash memory. It provides a good data calculation performance because of its ability to work with the large number of floating calculation points of the multi-sensor system.

**Figure 2:**
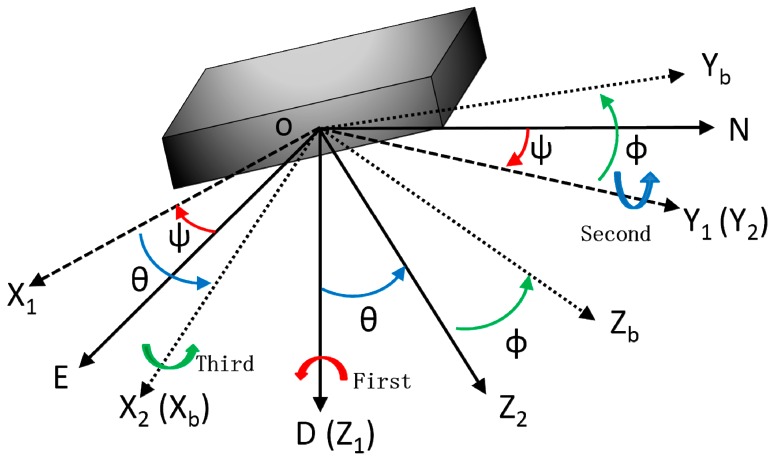
Transformation between NED and body coordinate system.

## 3. Coordinate System and Heading Estimation

### 3.1. Coordinate Systems

The main task of a wearable multi-sensor system is to determine attitude angles which are the main navigation parameters of a body segment. The designed multi-sensor system is based on the geographical coordinate system and body coordinate system shown in Figure 2. The geographical coordinate system determines the north, east and vertical directions of the position of the body. This coordinate system is linked to the local Earth referential [47]. The orthogonal axes of N, E and D represent the axis of north, east, and upright respectively. The body coordinate system is attached to the multi-sensor system, and X_b_, Y_b_ and Z_b_ represent the directions of its head, starboard and bottom respectively.

The body coordinate system (frame b) can be obtained from NED coordinate system (frame n) through three sequential rotation angles heading (ψ), pitch (θ) and roll (ϕ), which present the body attitude. The transformation matrix from ONED to OX_b_Y_b_Z_b_ is written as:

(1) $$C_{n}^{b}=\left[\begin{array}{ccc} \cos\psi \cos\theta & \sin\psi \cos\theta & -\sin\theta \\ -\sin\psi \cos\phi +\cos\psi \sin\theta \sin\phi & \cos\psi cos\phi +\sin\psi \sin\theta \sin\phi & \cos\theta \sin\phi \\ \sin\psi \sin\phi +\cos\psi \sin\theta \cos\phi & -\cos\psi \sin\phi +\sin\psi \sin\theta \cos\phi & \cos\theta \cos\phi \end{array}\right]$$

### 3.2. Heading Estimation

**Figure 3:**
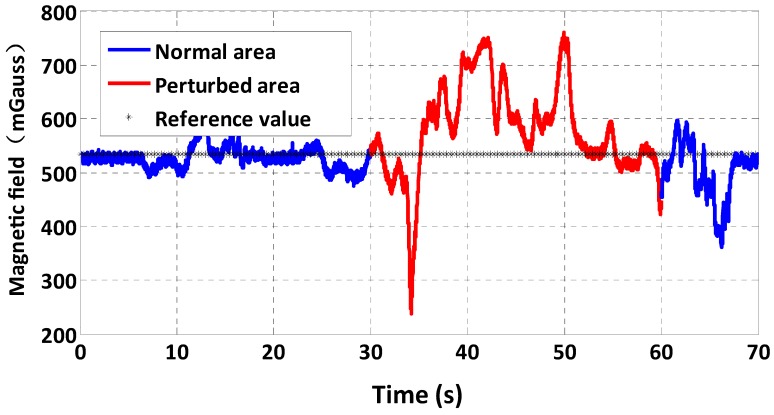
Indoor magnetic field test.

The magnetometer is easily subject to interferences from external magnetic sources such as the steel of buildings and electronic equipment. These kinds of disturbance lead to unpredictable performance of the magnetometer, which is a major drawback of using magnetic sensors. Figure 3 is an example of a test of the magnetometer of the multi-sensor system in a corridor of our college building. There are elevators and other metallic structures in the corridor which can produce magnetic sources to perturb the magnetometer, as illustrated by the red line in Figure 3. Therefore, magnetometer-based heading estimation is not reliable.

**Figure 4:**
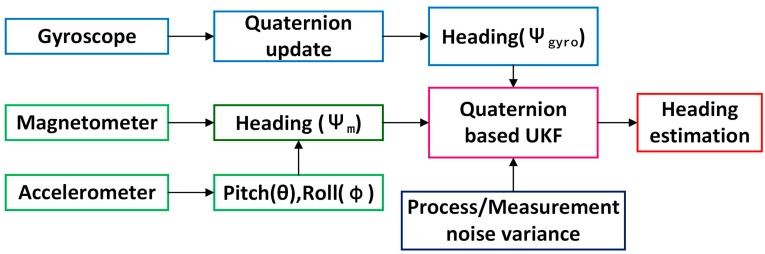
Flow chart of quaternion-based UKF for heading estimation.

However, the gyroscope is not perturbed by indoor magnetic disturbances. In addition, the magnetometer-based heading is relatively stable in tests over longer hours. A new heading estimation method is proposed to make use of complementary features of gyroscopes and magnetometers, meanwhile, the quaternion-based UKF to eliminate the errors derived from the gyroscope and magnetometer is adopted. The flow chart of accurate heading estimation using quaternion-based UKF is shown in Figure 4.

#### 3.2.1. Heading Estimation Using a Magnetometer

The pitch angle and roll angle can be computed from acceleration signals and the yaw angle is determining by computing the magnetometer output [48,49]. The relationship between gravitational acceleration components $\left(a_{x},a_{y},a_{z}\right)$ in the frame b and the gravity vector in the frame n, when regardless of its acceleration of multi-senor system, can be written as:

(2) $$\left[\begin{array}{c} a_{x}\\ a_{y}\\ a_{z} \end{array}\right]=C_{n}^{b}\left[\begin{array}{c} 0\\ 0\\ g \end{array}\right]=g\left[\begin{array}{c} -\sin\theta \\ \sin\phi \cos\theta \\ \cos\phi \cos\theta \end{array}\right]$$

With the acceleration outputs the pitch angle and roll angle can be obtained as:

(3) $$\left\{ \begin{array}{l} \theta =-\arctan(\frac{a_{x}}{\sqrt{a_{y}^{2}+a_{z}^{2}}})\\ \phi =\arctan(\frac{a_{y}}{a_{z}}) \end{array}\right.$$

However, the outputs of accelerometer are total accelerations, including the acceleration of the multi-senor system and the acceleration of gravity. In consideration of the multi-sensor system in indoor environments, GPS cannot be used in the acceleration measurement of the body coordinate system, meanwhile, the indoor body coordinate system is in a situation of low dynamics and motion at constant velocity. In this paper, the multi-sensor system fixed on pedestrians and quadrotors moves at a constant speed, and there is no sharp acceleration and deceleration in tests.

For heading angle computation with a magnetometer, the rotation relationship between the geomagnetic field intensity $\left(h_{x},h_{y},h_{z}\right)$ measured by the magnetometer in the frame b and the geomagnetic field intensity $\left(H_{x},H_{y},H_{z}\right)$ in the frame n could expressed as:

(4) $$\left[\begin{array}{c} H_{x}\\ H_{y}\\ H_{z} \end{array}\right]\text{=}\left[\begin{array}{ccc} \cos\theta & \sin\phi \sin\theta & \cos\phi \sin\theta \\ 0 & \cos\phi & -\sin\phi \\ -\sin\theta & \sin\phi \cos\theta & \cos\phi \cos\theta \end{array}\right]\left[\begin{array}{c} h_{x}\\ h_{y}\\ h_{z} \end{array}\right]$$

Considering the local magnetic declination, the real local heading estimation based on magnetometer is:

(5) $$\psi_{mag}=\arctan\left(\frac{-h_{y}\cos\phi +h_{z}\sin\phi }{h_{x}\cos\theta +h_{y}\sin\phi \sin\theta +h_{z}\cos\phi \sin\theta }\right)+D=\arctan\left(\frac{H_{2}}{H_{1}}\right)+D$$

where *D* is the local declination angle.

#### 3.2.2. Heading Estimation Using a Gyroscope

Quaternion has the advantage of no singularity when the pitch is approaching 90° and higher computation efficiency than Euler angles. Quaternion is a four-dimension vector and subject to normalization, which is defined as follows:

(6) $$q={\left[\begin{array}{cccc} q_{0} & q_{1} & q_{2} & q_{3} \end{array}\right]}^{T},\;q_{0}^{2}+q_{1}^{2}+q_{2}^{2}+q_{3}^{2}=1$$

Quaternion-based state update model with gyroscope measurement is shown as follows:

(7) $$\dot{q}=\frac{1}{2}q⊗\left[\begin{array}{c} 0\\ \omega_{x}\\ \omega_{y}\\ \omega_{z} \end{array}\right]=\frac{1}{2}\left[\begin{array}{cccc} 0 & -\omega_{x} & -\omega_{y} & -\omega_{z}\\ \omega_{x} & 0 & \omega_{z} & -\omega_{y}\\ \omega_{y} & -\omega_{z} & 0 & \omega_{x}\\ \omega_{z} & \omega_{y} & -\omega_{x} & 0 \end{array}\right]\left[\begin{array}{c} q_{0}\\ q_{1}\\ q_{2}\\ q_{3} \end{array}\right]$$

where $\omega_{x},\omega_{y},\omega_{z}$ are the measurements of gyroscope.

We could provide the analytical solution of Equation (7), and the discrete form is:

(8) $$q_{k+1}=[\cos(\frac{\vartheta }{2})I+\frac{\sin(\frac{\vartheta }{2})}{\frac{\vartheta }{2}}\cdot A\cdot dt]q_{k}$$

where *k* = 1, 2,…n, $A=\frac{1}{2}\left[\begin{array}{cccc} 0 & -\omega_{x} & -\omega_{y} & -\omega_{z}\\ \omega_{x} & 0 & \omega_{z} & -\omega_{y}\\ \omega_{y} & -\omega_{z} & 0 & \omega_{x}\\ \omega_{z} & \omega_{y} & -\omega_{x} & 0 \end{array}\right]$, $\vartheta =\sqrt{{\left(\omega_{x}\cdot dt\right)}^{2}+{\left(\omega_{y}\cdot dt\right)}^{2}+{\left(\omega_{z}\cdot dt\right)}^{2}}$, and *dt* is sampling time.

The quaternion-based traditional matrix from NED coordinate system to body coordinate system in DCM form is written as:

(9) $$C_{b}^{n}=\left[\begin{array}{ccc} q_{0}^{2}+q_{1}^{2}-q_{2}^{2}-q_{3}^{2} & 2(q_{1}q_{2}-q_{0}q_{3}) & 2(q_{1}q_{3}+q_{0}q_{2})\\ 2(q_{1}q_{2}+q_{0}q_{3}) & q_{0}^{2}-q_{1}^{2}+q_{2}^{2}-q_{3}^{2} & 2(q_{2}q_{3}+q_{0}q_{1})\\ 2(q_{1}q_{3}+q_{0}q_{2}) & 2(q_{2}q_{3}+q_{0}q_{1}) & q_{0}^{2}-q_{1}^{2}-q_{2}^{2}+q_{3}^{2} \end{array}\right]\text{ =}\left[\begin{array}{ccc} C_{11} & C_{12} & C_{13}\\ C_{21} & C_{22} & C_{23}\\ C_{31} & C_{32} & C_{33} \end{array}\right]$$

The reverse transformation matrix $C_{\text{n}}^{b}$ can be simply obtained as the transpose of $C_{b}^{n}$. Therefore, the attitude angles based on quaternion and gyroscope in form of Euler angles is expressed as:

(10) $$\{\begin{array}{c} \phi =\arctan(\frac{C_{32}}{C_{33}})\\ \theta =\arcsin(-C_{31})\\ \psi =\arctan(\frac{C_{21}}{C_{11}}) \end{array}\to \psi_{gyro}=\arctan(\frac{2(q_{1}q_{2}+q_{0}q_{3})}{q_{0}^{2}+q_{1}^{2}-q_{2}^{2}-q_{3}^{2}})$$

## 4. Quaternion-Based UKF

The Kalman filter is a model-based estimation technique. In this paper, the unscented Kalman filter is implemented to integrate the attitude determination from the gyroscope and magnetometer outputs through the quaternion method.

### 4.1. Kalman Filter Design

The state model *X* and measurement model *Y* are built respectively as follows:

(11) $$X={\left[\begin{array}{cccc} q_{0} & q_{1} & q_{2} & q_{3} \end{array}\right]}^{T}\text{, }Y={\left[\begin{array}{ccc} \phi & \theta & \psi \end{array}\right]}^{T}$$

The discrete time representation of Kalman filter is shown as:

(12) X(n+1)=F(n)X(n)+w(n)Y(n)=H[X(n)]+v(n)

where the system matrix *F* and output function H[X(n)] are:

(13) $$F=\cos(\frac{\vartheta }{2})\cdot I+\frac{\sin(\frac{\vartheta }{2})}{\frac{\vartheta }{2}}\cdot A\cdot dt$$

(14) $$H[X(n)]=\left[\begin{array}{l} \arctan\frac{2(q_{0}q_{1}+q_{2}q_{3})}{q_{0}^{2}-q_{1}^{2}-q_{2}^{2}+q_{3}^{2}}\\ \;\arcsin2(-q_{0}q_{2}-q_{1}q_{3})\\ \arctan\frac{2(q_{0}q_{3}+q_{1}q_{2})}{q_{0}^{2}+q_{1}^{2}-q_{2}^{2}-q_{3}^{2}} \end{array}\right]$$

*W* and *v* are the process noise and measurement noise, respectively. They are simplified as independent Gaussian white noise. The process noise covariance matrix and measurement noise covariance matrix are *Q* and *R* respectively.

### 4.2. Covariance of Process Noise and Measurement Noise

In the Kalman filter design, we consider the process noise and measurement noise as design parameters to achieve minimum for covariance of error estimation. Therefore, it is essential to determine the covariance of process noise and measurement noise. For heading estimation in this paper, the process noise derives from outputs ($\omega_{x}$, $\omega_{y}$, $\omega_{z}$) of MEMS gyroscopes, and we assume that $\omega_{x}={\bar{\omega }}_{x}+{\tilde{\omega }}_{x}$, $\omega_{y}={\bar{\omega }}_{y}+{\tilde{\omega }}_{y}$ and $\omega_{z}={\bar{\omega }}_{z}+{\tilde{\omega }}_{z}$, and ${\bar{\omega }}_{x}$, ${\bar{\omega }}_{y}$ and ${\bar{\omega }}_{z}$ are the mean of $\omega_{x}$, $\omega_{y}$ and $\omega_{z}$, and ${\tilde{\omega }}_{x}$, ${\tilde{\omega }}_{y}$ and ${\tilde{\omega }}_{z}$ are deviations of $\omega_{x}$, $\omega_{y}$ and $\omega_{z}$. The Equation (7) can be rewritten as [50]:

(15) $$\begin{array}{l} \left[\begin{array}{c} {\dot{q}}_{0}\\ {\dot{q}}_{1}\\ {\dot{q}}_{2}\\ {\dot{q}}_{3} \end{array}\right]=\frac{1}{2}\cdot \left[\begin{array}{cccc} 0 & -({\bar{\omega }}_{x}+{\tilde{\omega }}_{x}) & -({\bar{\omega }}_{y}+{\tilde{\omega }}_{y}) & -({\bar{\omega }}_{z}+{\tilde{\omega }}_{z})\\ \left({\bar{\omega }}_{x}+{\tilde{\omega }}_{x}\right) & 0 & \left({\bar{\omega }}_{z}+{\tilde{\omega }}_{z}\right) & -({\bar{\omega }}_{y}+{\tilde{\omega }}_{y})\\ \left({\bar{\omega }}_{y}+{\tilde{\omega }}_{y}\right) & -({\bar{\omega }}_{z}+{\tilde{\omega }}_{z}) & 0 & \left({\bar{\omega }}_{x}+{\tilde{\omega }}_{x}\right)\\ \left({\bar{\omega }}_{z}+{\tilde{\omega }}_{z}\right) & \left({\bar{\omega }}_{y}+{\tilde{\omega }}_{y}\right) & -({\bar{\omega }}_{x}+{\tilde{\omega }}_{x}) & 0 \end{array}\right]\left[\begin{array}{c} q_{0}\\ q_{1}\\ q_{2}\\ q_{3} \end{array}\right]\\ \text{ =}\frac{1}{2}\cdot \left[\begin{array}{cccc} 0 & -{\bar{\omega }}_{x} & -{\bar{\omega }}_{y} & -{\bar{\omega }}_{z}\\ {\bar{\omega }}_{x} & 0 & {\bar{\omega }}_{z} & -{\bar{\omega }}_{y}\\ {\bar{\omega }}_{y} & -{\bar{\omega }}_{z} & 0 & {\bar{\omega }}_{x}\\ {\bar{\omega }}_{z} & {\bar{\omega }}_{y} & -{\bar{\omega }}_{x} & 0 \end{array}\right]\left[\begin{array}{c} q_{0}\\ q_{1}\\ q_{2}\\ q_{3} \end{array}\right]+\frac{1}{2}\cdot \left[\begin{array}{ccc} -q_{1} & -q_{2} & -q_{3}\\ q_{0} & -q_{3} & -q_{2}\\ q_{3} & q_{0} & -q_{1}\\ -q_{2} & q_{1} & q_{0} \end{array}\right]\left[\begin{array}{c} {\tilde{\omega }}_{x}(t)\\ {\tilde{\omega }}_{y}(t)\\ {\tilde{\omega }}_{z}(t) \end{array}\right] \end{array}$$

The right term of Equation (15) can be regarded as process noise. For the discrete system, the covariance of process noise *Q* is:

(16) $$Q=\frac{1}{4}\left[\begin{array}{ccc} -q_{1} & -q_{2} & -q_{3}\\ q_{0} & -q_{3} & q_{2}\\ q_{3} & q_{0} & -q_{1}\\ -q_{2} & q_{1} & q_{0} \end{array}\right]\left[\begin{array}{ccc} \sigma_{\omega_{x}}^{2} & 0 & 0\\ 0 & \sigma_{\omega_{y}}^{2} & 0\\ 0 & 0 & \sigma_{\omega_{z}}^{2} \end{array}\right]{\left[\begin{array}{ccc} -q_{1} & -q_{2} & -q_{3}\\ q_{0} & -q_{3} & q_{2}\\ q_{3} & q_{0} & -q_{1}\\ -q_{2} & q_{1} & q_{0} \end{array}\right]}^{T}$$

where $\sigma_{\omega_{x}}^{2}$, $\sigma_{\omega_{y}}^{2}$ and $\sigma_{\omega_{z}}^{2}$ are variance of gyroscope outputs $\omega_{x}$, $\omega_{y}$ and $\omega_{z}$.

The covariance of measurement noise is decided by the measurement process. In this paper, the pitch (θ), roll (φ) and heading (ψ) angles are calculated from accelerometer and magnetometer outputs. We assume that, and ${\tilde{a}}_{x}$, ${\tilde{a}}_{y}$ and ${\tilde{a}}_{z}$ are deviation of accelerometer outputs $a_{x}$, $a_{y}$ and $a_{z}$. Through the Equation (2) and a Taylor series expansion, the covariance of pitch (θ) and roll (φ) can be derived as:

(17) $$\left[\begin{array}{c} \tilde{\phi }\\ \tilde{\theta } \end{array}\right]=\left[\begin{array}{ccc} 0 & \frac{a_{z}}{a_{y}^{2}+a_{z}^{2}} & -\frac{a_{y}}{a_{y}^{2}+a_{z}^{2}}\\ -\frac{\sqrt{a_{y}^{2}+a_{z}^{2}}}{a_{x}^{2}+a_{y}^{2}+a_{z}^{2}} & \frac{a_{x}a_{y}}{\left(a_{x}^{2}+a_{y}^{2}+a_{z}^{2}\right)\sqrt{a_{y}^{2}+a_{z}^{2}}} & \frac{a_{x}a_{z}}{\left(a_{x}^{2}+a_{y}^{2}+a_{z}^{2}\right)\sqrt{a_{y}^{2}+a_{z}^{2}}} \end{array}\right]\left[\begin{array}{c} {\tilde{a}}_{x}\\ {\tilde{a}}_{y}\\ {\tilde{a}}_{z} \end{array}\right]=M_{\phi \theta }\left[\begin{array}{c} {\tilde{a}}_{x}\\ {\tilde{a}}_{y}\\ {\tilde{a}}_{z} \end{array}\right]$$

With the $\sigma_{a_{x}}^{2}$, $\sigma_{a_{y}}^{2}$ and $\sigma_{a_{z}}^{2}$ are the variance of accelerometer $a_{x}$, $a_{y}$ and $a_{z}$, there is:

(18) $$R_{\phi \theta }=diag\left[\begin{array}{ccc} \sigma_{a_{x}}^{2} & \sigma_{a_{y}}^{2} & \sigma_{a_{z}}^{2} \end{array}\right]$$

The covariance of pitch and roll is as follows:

(19) $$\sigma_{\phi \theta }^{2}=M_{\phi \theta }R_{\phi \theta }M_{\phi \theta }^{T}$$

Similarly, the deviation of magnetometer based heading can be gotten from Equations (3) and (5) in a Taylor series expansion:

(20) $${\tilde{\psi }}_{mag}=\left[\begin{array}{cc} -\frac{H_{2}}{H_{1}^{2}+H_{2}^{2}} & \frac{H_{1}}{H_{1}^{2}+H_{2}^{2}} \end{array}\right]\left[\begin{array}{ccc} \cos\theta & \sin\theta \sin\phi & \cos\phi \sin\theta \\ 0 & -\cos\phi & \sin\phi \end{array}\right]\left[\begin{array}{c} {\tilde{h}}_{x}\\ {\tilde{h}}_{y}\\ {\tilde{h}}_{z} \end{array}\right]\text{=}M_{\psi }\left[\begin{array}{c} {\tilde{h}}_{x}\\ {\tilde{h}}_{y}\\ {\tilde{h}}_{z} \end{array}\right]$$

The variance of magnetometer based heading ($\psi_{mag}$) is expressed as:

(21) $$\sigma_{\psi }^{2}=M_{\psi }R_{\psi }M_{\psi }^{T}$$

where $R_{\psi }=diag\left[\begin{array}{ccc} \sigma_{h_{x}}^{2} & \sigma_{h_{x}}^{2} & \sigma_{h_{x}}^{2} \end{array}\right]$, and $\sigma_{h_{x}}^{2}$, $\sigma_{h_{y}}^{2}$ and $\sigma_{h_{z}}^{2}$ are variance of magnetometer outputs $h_{x}$, $h_{y}$ and $h_{z}$.

Finally, we can determine the covariance of measurement noise matrix *R* as:

(22) $$R=\left[\begin{array}{ccc} \sigma_{\theta \phi }^{2} & & \\ & \sigma_{\psi }^{2} & \\ & & \sigma_{C}^{2} \end{array}\right]$$

where $\sigma_{C}^{2}$ is variance of quaternion normalization equation and its value is zero, however, in order to avoid the mathematical problem in calculation, and $\sigma_{C}^{2}$ is given a small value.

### 4.3. Unscented Transformation

The unscented transformation (UT) is an effective way to approximate how the mean and covariance of a random variable change when it undergoes a nonlinear transformation. Consider propagating random variable *x* (dimension L) through a nonlinear function *y* = h(*x*). Assume X has mean $\bar{x}$ and covariance *P_x_*. To calculate the statistics of *y*, we form a matrix χ of 2L + 1 sigma vector $\chi_{i}$ (with corresponding weights $W_{i}$), according to the following:

(23) $$\begin{array}{l} \chi_{0}=\bar{x}\\ \chi_{i}=\bar{x}+\sqrt{(L+\lambda )P},\;i=1:L\\ \chi_{i}=\bar{x}-\sqrt{(L+\lambda )P},\;i=L+1:2L \end{array}$$

(24) $$\begin{array}{l} W_{0}^{m}=\frac{\lambda }{L+\lambda }\\ W_{0}^{c}=\frac{\lambda }{L+\lambda }+(1-\alpha^{2}+\beta )\\ W_{i}^{m}=\frac{\lambda }{2(L+\lambda )},\;i=1:2L \end{array}$$

where $\lambda =\alpha^{2}(L+\kappa )-L$ is a scaling factor. Usually, α is set to a small positive value (e.g., 1e−3), κ is set to 0 and β is set for 2 for Gaussian distribution.

### 4.4. UKF Algorithm Equations

The UKF is more robust and accurate than EKF under realistic initial attitude-error conditions and large attitude change when dealing with highly nonlinear problem. The UKF algorithm details are presented as follows. First, we initialize with inertial quaternion value as follows:

(25) $$\begin{array}{l} {\hat{x}}_{0}=E\left[x_{0}\right]\\ P_{0}=E\left[(x_{0}-{\hat{x}}_{0}){\left(x_{0}-{\hat{x}}_{0}\right)}^{T}\right] \end{array}$$

Secondly, sigma points are computed by:

(26) $$\chi_{k-1}=\left[{\hat{x}}_{k-1},{\hat{x}}_{k-1}+\gamma \sqrt{P_{k-1}},{\hat{x}}_{k-1}-\gamma \sqrt{P_{k-1}}\right]$$

Thirdly, the state update equations are expressed as:

(27) $$\begin{array}{l} \chi_{k\backslash k-1}=F\left[\chi_{k-1},u_{k-1}\right]\\ {\hat{x}}_{k}^{-}=\sum_{i=0}^{2L}W_{i}^{m}\chi_{i,k|k-1}\\ P_{k}^{-}=\sum_{i=0}^{2L}W_{i}^{c}[\chi_{i,k|k-1}-{\hat{x}}_{k}^{-}]{\left[\chi_{i,k|k-1}-{\hat{x}}_{k}^{-}\right]}^{T}+Q\\ y_{k|k-1}=H\left[\chi_{k|k-1}\right]\\ {\hat{y}}_{k}^{-}=\sum_{i=0}^{2L}W_{i}^{m}y_{i,k|k-1} \end{array}$$

where *F* and *H* are the highly nonlinear equations of above sections, and *Q* is the covariance of process noise.

Finally, the measurement update equations are given as follows:

(28) $$\begin{array}{l} P_{{\bar{y}}_{k}{\bar{y}}_{k}}=\sum_{i=0}^{2L}W_{i}^{c}[y_{i,k|k-1}-{\bar{y}}_{k}^{-}]{\left[y_{i,k|k-1}-{\bar{y}}_{k}^{-}\right]}^{T}+R\\ P_{x_{k}y_{k}}=\sum_{i=0}^{2L}W_{i}^{c}[\chi_{i,k|k-1}-{\hat{x}}_{k}^{-}]{\left[y_{i,k|k-1}-{\bar{y}}_{k}^{-}\right]}^{T}\\ K_{k}=P_{x_{k}y_{k}}{P_{{\bar{y}}_{k}{\bar{y}}_{k}}}^{-1}\\ {\hat{x}}_{k}={\hat{x}}_{k}^{-}+K_{k}(y_{k}-{\hat{y}}_{k}^{-})\\ P_{k}=P_{k}^{-}-K_{k}P_{{\bar{y}}_{k}{\bar{y}}_{k}}K_{k}^{T} \end{array}$$

where *R* is the covariance of measurement noise, and *K* is the Kalman gain.

## 5. Experiments and Result Analysis

### 5.1. Two-Axis Turntable Test of Multi-Sensor System

**Figure 5:**
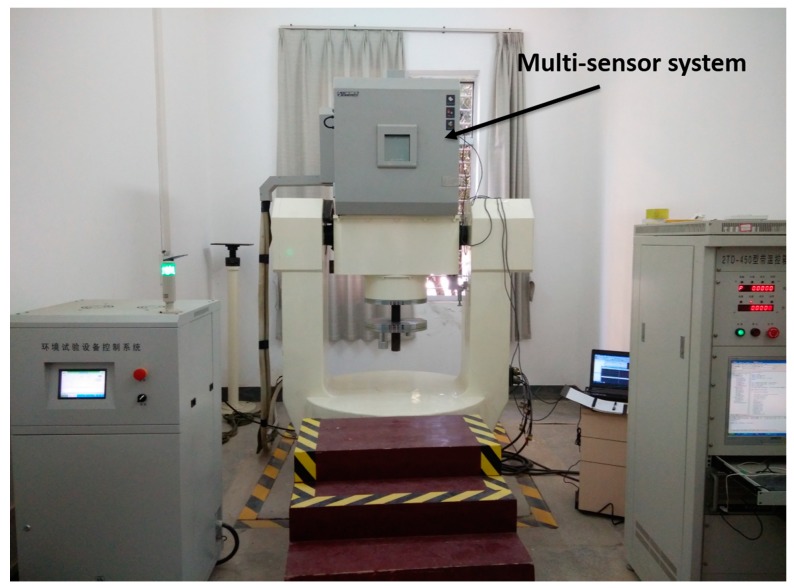
Two-axis turntable for testing multi-sensor system.

To evaluate the performance and measurement precision of our designed multi-sensor system, a two-axis turntable is used for static and dynamic test experiments. The two-axis turntable test platform is shown in Figure 5. The two-axis turntable is used not only for static and dynamic tests, but also for calibration tests of the multi-sensor system. Through test experiments including static, dynamic and heading determinations, the results show that our multi-sensor system with the proposed algorithm has a static accuracy better than 0.5°, dynamic accuracy better than 1° and heading accuracy better than 2°. Although the accuracy of our multi-sensor system is a little lower than the MTi-300 from Xsens [51], however, our multi-sensor system smaller and much cheaper than the MTi-300 so it is more suitable for wearable applications like indoor robots, UAVs, pedestrians and so on.

**Figure 6:**
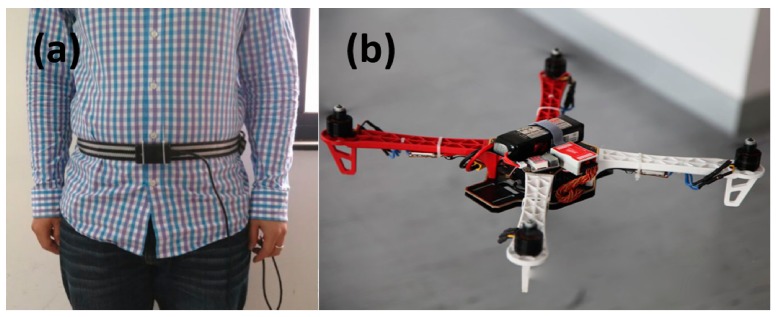
Heading estimation tests with the multi-sensor system (**a**) fixed on the waist of pedestrian; (**b**) fixed on a quadrotor UAV.

### 5.2. Indoor Heading Experiments

**Figure 7:**
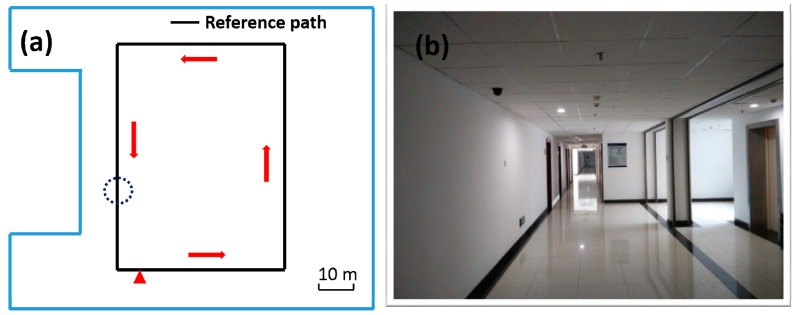
Heading estimation test floor plan (**a**) skeleton map; (**b**) picture of the actual corridor in the position of the circle in (a).

In order to verify that our wearable multi-sensor system using the quaternion-based UKF algorithm can meet the accurate heading estimation needs of robots, pedestrians and UAVs in indoor environments, we carried out two experiments in our college building corridor. One experiment is implemented with our multi-sensor system fixed on the waist of a pedestrian and the other one is carried out with our multi-sensor system fixed on a quadrotor UAV, as shown in Figure 6. The reference path of our experiments is the rectangular black line on the floor plan of our college building shown in Figure 7, and (a) is the skeleton map of floor plan; and (b) is a picture of the actual corridor from the position of the circle in (a). Both experiments are carried out strictly along the reference path in the red arrow direction. Each experiment starts from the red triangle and ends at the red triangle.

**Figure 8:**
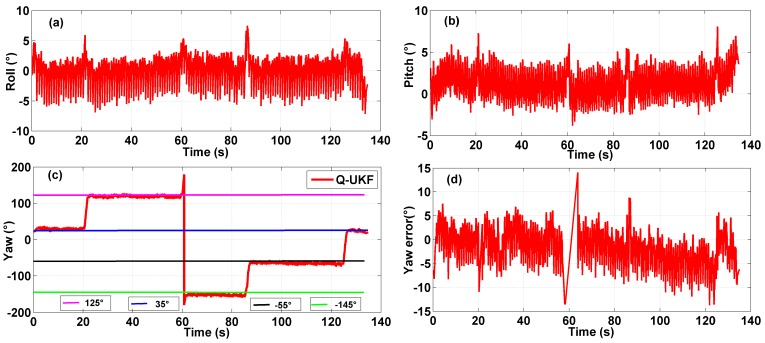
Multi-sensor system on waist of a pedestrian test: (**a**) roll angle; (**b**) pitch angle; (**c**) yaw angle; (**d**) yaw angle error.

### 5.3. Result Analysis

**Figure 9:**
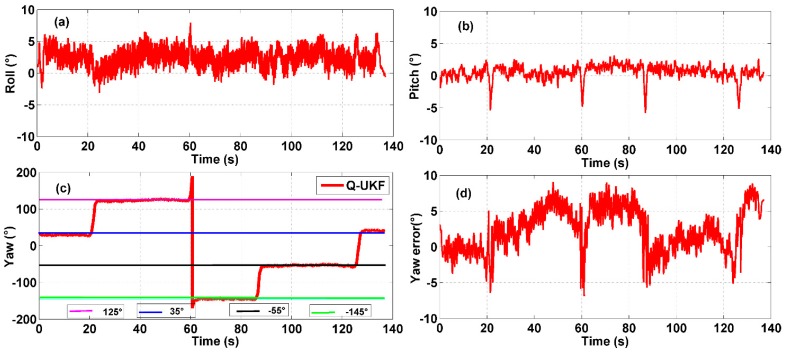
Multi-sensor system on a quadrotor test: (**a**) roll angle; (**b**) pitch angle; (**c**) yaw angle; (**d**) yaw angle error.

**Figure 10:**
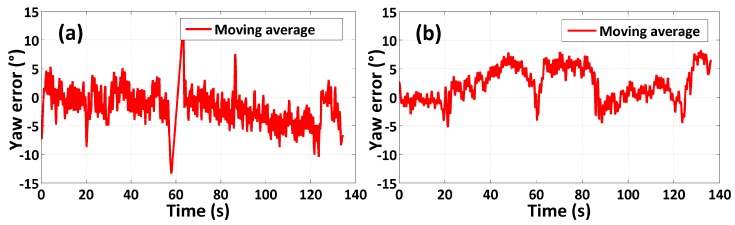
Moving average of yaw error: (**a**) multi-sensor system on the pedestrian test; (**b**) multi-sensor system on the quadrotor test.

The results of experiments using the multi-sensor system on the waist of a pedestrian and on a quadrotor UAV for indoor heading estimation using our proposed quaternion-based UKF algorithm are shown in Figure 8 and Figure 9, respectively, in which Figure 8 and Figure 9a–c are three attitude angles of the pedestrian and the quadrotor. In Figure 8 and Figure 9c, the red line is the heading estimation, and the four straight lines in pink, blue, black and green are reference path directions in the corridor of our college building. Figure 8 and Figure 9d are the error between the heading estimation using our proposed algorithm and the reference path directions. Figure 10 is the moving average of the yaw error analysis, which is a kind of technical analysis tool commonly used with time series data to smooth out short-term fluctuations and highlight longer-term trends. In this paper, we define the moving points as 50. By comparison with the evolution of the error and moving error average, it is found that the moving average method can be more informative than the evolution of the error.

It is easy to obtain the mean heading estimation error of the multi-sensor system on a pedestrian’s waist as about 10° by contrast with the reference path. The mean heading estimation error of the multi-sensor system on a quadrotor is about 5°. Because the heading range is from −180° to 180°, the rates of mean heading errors are 5.56% (10°/180°) and 2.78% (5°/180°) Due to the oscillation in all tests, an error rate ≤5% can be identified as an accurate indoor heading estimation. Through comparison of Figure 8 and Figure 9, we can obviously find out that the roll angle and pitch angle of the multi-sensor system on pedestrian’s waist have a larger variation range than that of the multi-sensor system on the quadrotor, which indicates the maneuvering conditions of the quadrotor are more stable than those of pedestrian. Nonetheless, the heading estimation error of the quadrotor has more dynamic changes than that of the pedestrian, which means there is external perturbation affecting the heading estimation. From an analysis of the indoor disturbance sources and a comparison between the pedestrian and quadrotor data mentioned above, it is found that the four brushless electric machines of the quadrotor can produce a certain external magnetic disturbance to have an influence on the heading estimation, which however is difficult to eliminate.

Although the experiments have verified that heading estimation with our wearable multi-sensor system using quaternion-based unscented Kalman filter has high accuracy, however, in the experiments the pedestrian and quadrotor are set into motion at a constant velocity, which can guarantee an accurate heading estimation. In practice however, the indoor movement conditions are complex, especially when emergency events occur. Therefore, experiments under complicated conditions should be carried out along to improve the heading estimation algorithm.

## 6. Conclusions

In this paper, a wearable multi-sensor system based on nine degrees of freedom low-cost MEMS sensors and STM32F407 has small size and good cost performance. The proposed quaternion-based unscented Kalman filter can use the complementary features of gyroscopes and magnetometers to get accurate heading estimations. It was tested through indoor experiments with the multi-sensor system fixed on the waist of a pedestrian and a quadrotor UAV, and the results show that the mean heading estimation errors are about 10° and 5°, respectively, in comparison to the reference path. However, in the future, we will carry out experiments in more complicated conditions such as sharp acceleration and deceleration, and in the next step we will combine the accurate heading estimation with distance estimation, the other element of indoor dead-reckoning, for accurately navigating robots, pedestrians and UAVs. The WiFi technique and camera technique are other promising indoor navigation techniques. We will combine WiFi or a camera with the IMU, and carry out feature recognition studies of buildings to calibrate the multi-sensor system in order to improve the location accuracy.
